# UTILIZING TECHNOLOGY NETWORKS TO SUPPORT SOCIAL NETWORKS FOR PEOPLE AGING WITH DISABILITY

**DOI:** 10.1093/geroni/igz038.2787

**Published:** 2019-11-08

**Authors:** Elena T Remillard, Wendy Rogers, Sarah Ruiz

**Affiliations:** 1 Georgia Institute of Technology, Atlanta, Georgia, United States; 2 University of Illinois, Urbana-Champaign, Champaign, Illinois, United States; 3 National Institute on Disability, Independent Living, and Rehabilitation Research, Washington, D.C., United States

## Abstract

A growing number of new smart, internet-enabled technologies from smart phone applications, to teleconferencing, to the Internet of Things (IoT), provide great promise and potential to support successful aging-in-place for people with long-term disabilities. This symposium highlights ongoing research at the TechSAge Rehabilitation Engineering Research Center to identify technology needs and develop/adapt new technologies to promote independence, health, and participation of this population. To understand user needs, Harris et al. will present findings from a large-scale interview study with older adults with long-term vision and mobility disabilities (N=120) that explored specific task-based challenges with community activities (e.g., going to entertainment events, volunteering) as well as solutions and strategies to overcome them. Koon et al. will present findings on perceived facilitators and barriers to using digital assistants (e.g., Amazon Alexa) to facilitate a variety of everyday tasks at home, from shopping to communicating with others, among adults aging with mobility disabilities. Levy et al. will discuss findings from research driving the creation of augmented reality tools that can enable individuals to experience how IoT devices, such as smart thermostats and lightbulbs, could be used within the context of one’s own abilities and home. Mitzner et al., will describe the development of a Tele Tai Chi intervention for older adults with long-term mobility disabilities that employs teleconferencing software to translate an in-person, evidence-based class to an online, social experience. TechSAge Program Officer, Sarah Ruiz (National Institute on Disability, Independent Living, and Rehabilitation Research), will serve as the discussant.

